# Stability Indicating HPLC Method for the Determination of Chiral Purity of R-(-)-5-[2-aminopropyl]-2-methoxybenzene Sulfonamide

**DOI:** 10.4103/0250-474X.58187

**Published:** 2009

**Authors:** G. B. Kasawar, M. N. Farooqui

**Affiliations:** Dr. Rafiq Zakaria Campus, Post graduate Studies and Research Centre, Moulana Azad College, Rouza Bagh, Aurangabad-431 001, India

**Keywords:** Tamsulosin hydrochloride, R-(-)-5-[2-aminopropyl]-2-methoxybenzene sulfonamide, chiral purity, validation, active pharmaceutical ingredient

## Abstract

A chiral reverse phase liquid chromatographic method was developed for the enantiomeric resolution of racemic mixture of (-)-5-[2-aminopropyl]-2-methoxybenzene sulfonamide in bulk drug. The enantiomeric separation of sulfonamide was resolved on a Crownpak CR (+) column using perchloric acid buffer of pH 1.0 as mobile phase and with UV detection at 226 nm. The method is validated and proved to be robust. The limit of detection and quantification of S (-)-(5)-[2-aminopropyl]-2-methoxybenzene sulfonamide] was found to be 0.084 and 0.159 μg/ml, respectively for 20 μl injection volume. The percentage recovery of S (-)-(5)-[2-aminopropyl]-2-methoxybenzene sulfonamide] ranged from 99.57 to 101.88 in bulk drug samples of R (-)-(5)-[2- aminopropyl]-2-methoxybenzene sulfonamide].

In continuation of our previous work[1–3] on chromatographic stability indicating parameters of drug, we decided to study stability indicating HPLC determination of chiral purity of R-(-)-5-[2-aminopropyl]-2-methoxybenzene sulfonamide, (R-(-)-(5)-[2-APMBS], fig. 1). This isomer is used in the synthesis of tamsulosin hydrochloride (fig. 1). The chiral purity of drug is very important since only R-isomer of it is active and S-tamsulosin is inactive. Tamsulosin is a selective antagonist at α-1A and α-1B adrenoceptors and exhibits enhanced specificity for the receptors in prostate and is commonly used to treat benign prostatic hyperplasia[4]. Literature survey reveals that few workers reported the chiral purity of tamsulosin by HPLC[5] and by CE,[6,7] but it is desirable to check the chiral purity of intermediates of a drug in its synthetic route. Hence we decided to determine the chiral purity of R (-)-(5)-[2-APMBS] a product at penultimate step in the synthetic route of tamsulosin hydrochloride.

**Fig. 1:**
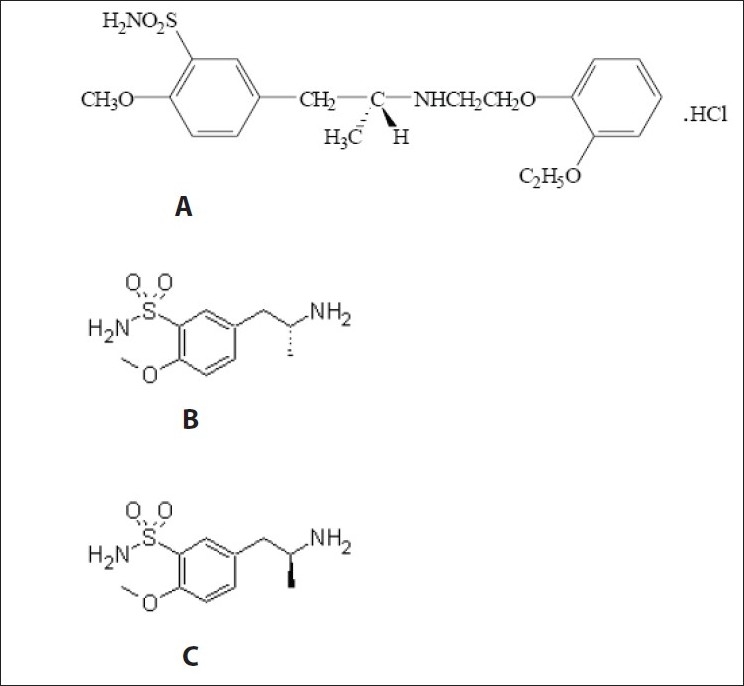
Chemical structures of Tamsulosin HCl and pure enantiomers A. Tamsulosin HCl, B. R-(-)-5-[2-aminopropyl]-2-methoxybenzene sulfonamide and C. S-(+)-5-[2-aminopropyl]-2-methoxybenzene sulfonamide

## MATERIALS AND METHODS

R (-)-(5)-[2-APMBS] and S (-)-(5)-[2-APMBS] were supplied by Research and Development department of Wockhardt limited, Aurangabad, India. Perchloric acid (Exelar grade) was purchased from Qualigens, India. Water used for the study was from Milli-Q system (Millipore, Bedford, MA, USA).

The HPLC used was a Waters 2690 series LC system with photo diode array detector and inbuilt auto injector (Alliance) was used for method development and validation. Waters Empower software was used for data acquisition and system suitability calculations. The columns used were Chiral AGP (Chrom Tech), Chiralcel OD (Diacel), Chiralpak AD (Diacel), YMC Chiral NEA (R) and Crownpak CR (+).

### Chromatographic Conditions:

The chromatographic conditions were optimized using Crownpak CR (+) (150 × 4.0 mm, 5 μ, (Chiral Technologies, Europe) column containing a chiral crown ether as a chiral selector. Water adjusted to pH 1.0 with perchloric acid was used as mobile phase with 0.5 ml/min flow. The column was thermostated in column oven at 30° and the UV detection was carried out at wavelength of 226 nm. The injection volume of 20 μl was used. The analyte concentration of R (-)-(5)-[2-APMBS] was fixed as 1 mg/ml. The working solution of R (-)-(5)-[2-APMBS] and its (S)-enantiomer was prepared in mobile phase. The developed method was validated according to the ICH guidelines[8].

### Specificity:

**Fig. 2:**
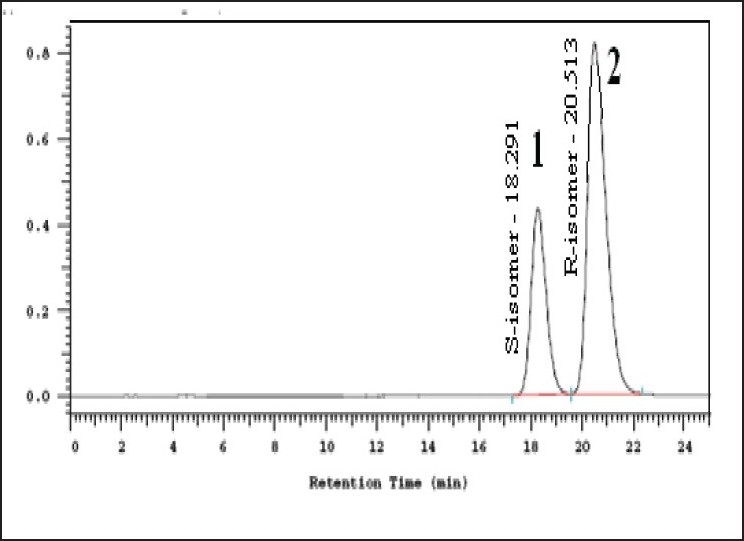
Chromatogram showing resolution of S and R-enantiomers 1. S-(-)-5-[2-aminopropyl]-2-methoxybenzene sulfonamide and 2. R-(+)-5-[2-aminopropyl]-2-methoxybenzene sulfonamide

The specificity of the developed LC method for R (-)-(5)-[2-APMBS] was determined in the presence of its impurities and degradation products. Forced degradation studies were also performed on R (-)-(5)-[2-APMBS] to provide an indication of the stability indicating property and specificity of the proposed method. The stress conditions employed for degradation study includes light (2600 Lux), heat (105°), humidity (25°/95% RH), acid hydrolysis (5N HCl), base hydrolysis (2N NaOH) and oxidation (30% w/v H_2_O_2_). For light, heat and humidity studies, sample was exposed for about 95 h. The typical chromatograms of control sample and stressed samples are represented in fig. 2. About 9% degradation was found in peroxide treated sample showing few degradation products which are not interfering with main drug. In other stressed conditions degradation has not been seen. Peak purity of stress sample of R (-)-(5)-[2-APMBS] was checked by using photo diode array detector (PDA). Purity angle is less than purity threshold in all stress samples and no interference from blank demonstrates the analyte peak homogeneity.

### Method reproducibility:

Method reproducibility was determined by measuring repeatability and intermediate precision of peak area counts of each enantiomer. In order to determine the repeatability of the method, replicate injection (*n*=6) of a 1.0 mg/ml solution containing R (-)-(5)-[2-APMBS] with (S)-enantiomer (0.2%) was carried out. The intermediate precision was also evaluated on two different days by performing six successive injections each day.

### Limit of detection and limit of quantification of (S)-enantiomer:

The limit of detection (LOD) is estimated as three times the signal to noise ratio and the limit of quantification (LOQ) is estimated as ten times the signal to noise ratio[8]. LOD and LOQ were achieved by injecting a series of dilute solutions of (S)-enantiomer. The precision of the developed chiral method for (S)-enantiomer at limit of quantification was checked by analyzing six test solution of (S)-enantiomer prepared at LOQ level and determined the percentage relative standard deviation of peak area.

### Linearity of (S)-enantiomer:

Detector response linearity was assessed by preparing 12 different calibration solutions of (S)-enantiomer covering concentration range from 0.064 μg/ml to 6.378 μg/ml in mobile phase from its stock solution. Regression curve was obtained by plotting peak area versus concentration, using the least squares method. The slope and intercept of the calibration curve was calculated. The linear regression equation is y=51176 X–438 and R^2^ is 0.99996.

### Quantification of (S)-enantiomer in bulk sample:

R (-)-(5)-[2-APMBS] bulk sample supplied showed absence of (S)-enantiomer. Standard addition and recovery experiment were conducted to determine the accuracy of the present method for the quantification of (S)-isomer in bulk drug sample. The study was carried out in triplicate at 0.10, 0.20 and 0.25% of R (-)-(5)-[2-APMBS] target analyte concentration.

### Robustness:

To determine the robustness of the method, experimental conditions were deliberately altered and chromatographic resolution between R (-)-(5)-[2-APMBS] and (S)-enantiomer was evaluated. The flow rate of the mobile phase was 0.5 ml/min. To study the effect of flow rate on the resolution of enantiomer, a range of 0.4 to 0.6 ml/min is used. The effect of change in pH of mobile phase was studied by varying from pH 0.8 to 1.2. The effect of column oven temperature on resolution was studied at 25° and 35° instead of 30° while other chromatographic components were held constant as stated in section 2.4.

### Solution stability:

Stability of R (-)-(5)-[2-APMBS] in solution at analyte concentration was studied by keeping the solution in tight capped volumetric flask at room temperature on a laboratory bench for 16 h. Content of (S)-enantiomer was checked for every hour interval.

## RESULTS AND DISCUSSION

The aim of this work is to separate the enantiomers of R (-)-(5)-[2-APMBS] and accurate quantification of (S) enantiomer. Racemic mixture solution of 0.5 mg/ml prepared in mobile phase was used in method development. To develop a rugged and suitable LC method, different mobile phases and stationary phases were employed. Various experiments were conducted using various chiral columns to select best stationary and mobile phase that would give optimum resolution and better selectivity for the two enantiomers. Separation was not found on Chiral AGP, Chiralcel OD, Chiralpak AD and YMC Chiral NEA (R) columns using different possible mobile phases. There is an indication of separation on Crownpak CR (+) column using perchloric acid buffer as mobile phase. Crownpak CR (+) column contains chiral crown ether as a chiral selector. Its chiral recognition lies when a complex is formed between the crown ether and an ammonium ion obtained from primary amine of R (-)-(5)-[2-APMBS]. Mobile phase with lower pH range from 1 to 2 forms the quaternary ammonium ion of primary amines. Several acidic mobile phases such as nitric acid, trifluoroacetic acid and perchloric acid can be used in pH range 1 to 2. Perchloric acid is preferred as mobile phase due to low UV absorption. Decrease in pH of the mobile phase enhanced the chromatographic efficiency and resolution between the enantiomers. Very good separation was achieved on Crownpak CR (+) column (resolution between enantiomers was found greater than 2.5) using water adjusted to pH 1 with perchloric acid as a mobile phase system. Hence the method validation was carried out on the same.

In the optimized method, the typical retention time of (S)-enantiomer and R (-)-(5)-[2-APMBS] were about 18.6 and 21.2 min, respectively. The enantiomeric separation of R (-)-(5)-[2-APMBS] on Crownpak CR (+) column is shown in fig. 2. The system suitability test results of the chiral LC method are presented in Table 1.

**TABLE 1:** SYSTEM SUITABILITY REPORT

Compound	R_t_	R_s_	N	T
(S)-enantiomer	18.3		6023	1.2
(R)-enantiomer	20.5	2.8	6491	1.0

R_t_: retention time, R_s_: USP resolution, N: number of theoretical plates (USP tangent method), T: USP tailing factor.

In the repeatability study, the percentage relative standard deviation (%RSD) was <1% for the retention time of both the enantiomers, <1.5 % for the peak area counts for both the enantiomers and <5% for %w/w of (S)-enantiomer in drug substance. In intermediate precision study, results shows that RSD values in the same magnitude than those obtained for repeatability (Table 2).

**TABLE 2:** VALIDATION RESULTS

Validation parameters	Results
Repeatibility : n=6, %RSD	
Retention time (S)-enantiomer	0.6
Retention time (R)-enantiomer	0.7
Peak area (S)-enantiomer	1.3
Peak area (R)-enantiomer	1.1
% w/w of (S)-enantiomer	3.5
Intermediate Precision : n=12, %RSD	
Retention time (S)-enantiomer	0.5
Retention time (R)-enantiomer	0.6
Peak area (S)-enantiomer	1.1
Peak area (R)-enantiomer	0.9
% w/w of (S)-enantiomer	3.2
LOD-LOQ : S-enantiomer	
Limit of detection (μg/ml)	0.084
Limit of quantification (μg/ml)	0.156
Precision at LOQ (%RSD)	8.2
Linearity : R-enantiomer	
Calibration range (μg/ml)	0.064-6.378
Calibration points	12
Correlation coefficient	0.99996

**Fig. 3:**
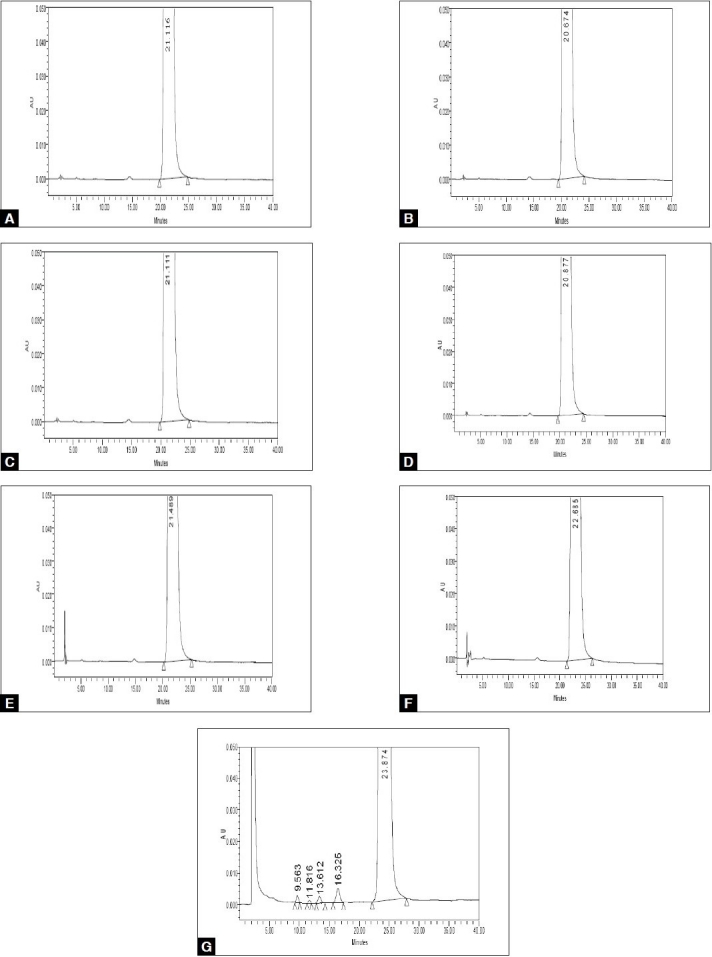
Typical HPLC chromatograms of pure and stressed samples A. Untreated sample, B. sample stressed under UV, C. sample stressed under heat. D. sample stressed under humidity, E. sample treated with acid, F. sample treated with base and G. sample treated with peroxide

The limit of detection (LOD) and quantification (LOQ) concentrations were estimated to be 0.084 and 0.156 μg/ml for (S)-enantiomer (Table 2). Linearity was observed for (S)-enantiomer over the concentration range of 0.064 to 6.378 μg/ml, with the linearity regression equation y=51176x–437 and R^2^ is 0.99996 (Table 2). Standard addition and recovery experiments were conducted for (S)-enantiomer in bulk sample in triplicate at 0.1, 0.2, 0.25% of analyte concentration and percentage recovery ranged from 99.57 to 101.88 (Table 3). The chromatographic resolution of R (-)-(5)-[2-APMBS] and (S)-enantiomer peak was used to evaluate the method robustness under modified conditions. The resolution between R (-)-(5)-[2-APMBS] and (S)-enantiomer was found greater than 1.5, under all modified conditions (Table 4), demonstrating sufficient robustness. No significant change in the (S)-enantiomer content was observed in R (-)-(5)-[2-APMBS] sample during solution stability experiment. Hence, R (-)-(5)-[2-APMBS] sample solution is stable for at least 16 h. The method was found to be stability-indicating as evidenced by the chromatograms generated after the sample has been subjected to various stress conditions (fig. 3)

**TABLE 3:** RECOVERY OF (S)-ENANTIOMER IN BULK DRUG

Amount added (μg/ml) (n=3)	Amount recovered (μg/ml)	Recovery (%)	RSD (%)
1.03	1.05	101.94	2.2
2.06	2.04	99.03	2.6
2.47	2.48	100.40	1.9

n=3 determination

**TABLE 4:** ROBUSTNESS OF CHIRAL LC METHOD

Variable Parameter		USP resolution between R-(-)-5 [2- APMBS] and (S)-enantiomer
Flow rate (ml/min):	0.4	1.9
	0.5	2.1
	0.6	1.8
Column temperature (°):		
	25	2.2
	30	2.0
	35	1.9
Mobile phase pH:		
	0.8	2.1
	1.0	2.3
	1.2	1.7

A simple, rapid and accurate reverse phase chiral LC method was described for the enantiomeric separation of R (-)-(5)-[2-APMBS]. Crownpak CR (+) column containing crown ether as a chiral selector was found to be selective for the enantiomers of R (-)-(5)-[2-APMBS]. The method was validated showing satisfactory data for all the method validation parameters tested. The developed method is stability indicating and can be used for the quantitative determination of chiral impurity (S)-enantiomer in bulk materials.
